# 复发/难治多发性骨髓瘤患者嵌合抗原受体T细胞治疗后造血恢复情况及影响因素分析

**DOI:** 10.3760/cma.j.issn.0253-2727.2022.10.010

**Published:** 2022-10

**Authors:** 婷婷 赵, 娇 葛, 宏 朱, 荣冰 马, 春雨 李, 重阳 万, 莹 王, 开林 徐, 振宇 李

**Affiliations:** 徐州医科大学附属医院血液科，徐州 221006 Department of Hematology, the Affiliated Hospital of Xuzhou Medical University, Xuzhou 221002, China

嵌合抗原受体T细胞（CAR-T）疗法在复发/难治多发性骨髓瘤（R/R MM）患者中已取得较为满意的疗效[1]–[3]。综合其他团队及我们前期的研究，CAR-T治疗后血液学毒性发生率较高[4]–[6]，部分患者血象恢复时间长，影响临床疗效。本研究我们着眼于探索CAR-T治疗后血液学毒性的发生规律及其恢复情况，并分析其影响因素，以期为早期预防提供参考。

## 病例与方法

1. 病例资料：回顾性分析2017年6月1日至2020年12月31日中国临床试验注册中心注册（ChiCTR-OIC-17011271与ChiCTR-OIC-17011272）的79例R/R MM患者资料，诊断均符合2016年国际骨髓瘤工作组（IMWG）诊断标准。收集患者年龄，性别，既往治疗经过，疾病分型和分期，CAR-T靶点的选择，CAR-T细胞输注时间及细胞数量，患者接受CAR-T细胞输注后30 d内的体温、脉搏、血压、脉氧等变化情况等临床资料；收集血常规、肝功能、肾功能、电解质、LDH、β_2_微球蛋白水平、外周血铁蛋白、IL-6、C反应蛋白（CRP）、骨髓浆细胞比例等实验室资料。研究获得徐州医科大学附属医院临床试验伦理委员会批准（批件号：XYFY2017-KL014-01、XYFY2017-KL013-01）。

2. CAR-T治疗方案：CAR-T细胞静脉输注前5 d开始进行FC方案预处理：氟达拉滨30 mg·m^−2^·d^−1^，−5～−3 d，联合环磷酰胺750 mg·m^−2^·d^−1^，−5 d。在第0天输注CAR-T细胞1×10^6^/kg。

3. 血液学毒性评估及造血恢复定义：血液学毒性指标参考CTCAE5.0不良反应评估标准进行评估，细胞因子释放综合征（CRS）分级参考文献[7]标准。参照文献[5],[8]定义，1周内不输血的情况下HGB>80 g/L、PLT>50×10^9^/L，2周内不使用G-CSF的情况下WBC>3.0×10^9^/L、ANC>1.0×10^9^/L，或HGB增加>15 g/L、PLT增加>20×10^9^/L，ANC增加>0.5×10^9^/L定义为血细胞恢复；HGB、PLT、WBC、ANC均恢复定义为完全恢复。

4. 统计学处理：采用SPSS 22.0进行统计学分析。根据28 d血细胞计数情况将患者分为完全恢复组与未完全恢复组，比较两组患者临床特征。等级资料及不符合正态分布的计量资料组间比较采用Mann-Whitney *U*检验，符合正态分布的计量资料组间比较采用独立样本*t*检验，无序分类资料组间比较采用Fisher确切概率法。单因素与多因素Logistic回归分析影响治疗后完全恢复的影响因素。*P*<0.05为差异有统计学意义。

## 结果

1. 患者基本情况：79例患者中，男51例（64.6％），女28例（35.4％），中位年龄58（30～70）岁。IgA型17例（21.5％），IgG型32例（40.5％），IgD型7例（8.9％），λ轻链型11例（13.9％），κ轻链型8例（10.1％），不分泌型4例（5.1％）；ISS分期：Ⅰ期16例（20.3％）、Ⅱ期30例（38.0％）、Ⅲ期33例（41.8％）；18例（22.8％）既往接受自体造血干细胞移植。CAR-T细胞靶点：CD19联合BCMA 45例（57.0％），BCMA 30例（38.0％），其他靶点4例（5.1％）；CAR-T细胞治疗后13例（16.5％）未发生CRS，57例（72.2％）发生1～2级CRS，9例（11.4％）发生3～4级CRS；治疗后最佳疗效评估达到缓解的77例（97.5％），未缓解2例（2.5％）；54例（68.4％）存在基线血细胞减少。53例（67.1％）患者接受了G-CSF或成分输血治疗，其中输注红细胞27例，输注血小板30例，接受G-CSF 53例。

2. 外周血细胞减少的严重程度：68.4％（54/79）的患者在预处理前即存在血细胞减少，大部分为轻度。预处理或输注CAR-T细胞后贫血78例（98.7％）、血小板减少67例（84.8％）、中性粒细胞减少76例（96.2％）、白细胞减少77例（97.5％），其中≥3级的分别为47例（59.5％）、46例（58.2％）、65例（82.3％）、68例（86.1％）。白细胞减少大多在预处理后即出现，而贫血及血小板减少发生的时间稍迟。WBC中位最低值为1.30×10^9^/L，ANC中位最低值为1.02×10^9^/L，HGB中位最低值为86 g/L，PLT中位最低值为57×10^9^/L。

3. 外周血细胞计数恢复：在输注CAR-T细胞后第28天时，25例（31.6％）WBC恢复，32例（40.5％）ANC恢复，45例（57.0％）HGB恢复，42（53.2％）例PLT恢复。第90天时，各系血细胞计数恢复比例增加，71.0％ WBC恢复，81.1％ ANC恢复，91.3％ HGB恢复，88.1％ PLT恢复。截至2021年8月，WBC、ANC、HGB、PLT恢复的中位时间分别为45（26～76）、41（19～67）、35（27～62）、37（24～66）d。

4. 完全恢复的影响因素分析：分析患者是否获得完全恢复的影响因素，结果见表1，IL-6、铁蛋白、CRP峰值及基线血细胞水平是获得完全恢复的影响因素。将上述因素进一步多因素分析，结果显示CRP峰值<68.8 mg/L（*OR*＝9.321，95％ *CI* 1.461～59.449，*P*＝0.018）、IL-6峰值>349 pg/L（*OR*＝53.911，95％ *CI* 4.144～701.346，*P*＝0.002）、基线ANC<1.78×10^9^/L（*OR*＝12.315，95％ *CI* 1.539～98.509，*P*＝0.018）、基线PLT<141×10^9^/L（*OR*＝32.407，95％ *CI* 4.264～246.313，*P*＝0.001）是影响完全恢复的独立危险因素。

**表1:** 影响嵌合抗原受体T细胞治疗在复发/难治多发性骨髓瘤患者血细胞计数完全恢复的单因素分析

因素	单因素		
	*OR*	95%*CI*	*P*值
年龄	0.958	0.890～1.030	0.243
治疗线数	1.278	0.975～1.674	0.075
自体移植	3.349	0.697～16.090	0.131
ISS分期			
Ⅰ期	参照		
Ⅱ期	0.917	0.228～3.684	0.902
Ⅲ期	1.042	0.261～4.155	0.954
靶点含BCMA	0.755	0.268～2.126	0.595
缓解	3.053	0.182～51.204	0.438
CRS分级	1.627	0.610～4.335	0.331
浆细胞比例	1.018	0.989～1.049	0.225
β_2_-MG≥5.5 mg/L	2.543	0.521～12.414	0.248
CRP峰值<68.8 mg/L	5.077	1.514～17.029	0.009
IL-6峰值>349 pg/L	13.971	1.752～111.394	0.013
铁蛋白峰值>1310 µg/L	3.538	1.212～10.328	0.021
基线WBC<4.05×10^9^/L	7.412	2.327～23.605	0.001
基线ANC<1.78×10^9^/L	9.964	2.120～46.837	0.004
基线HGB<103 g/L	6.316	2.000～19.947	0.002
基线PLT<141×10^9^/L	17.550	3.698～83.280	0.001

注：ISS分期：国际分期系统；CRS：细胞因子释放综合征；β_2_-MG：β_2_微球蛋白；CRP：C反应蛋白

## 讨论

CAR是一种基因修饰的跨膜蛋白，由一个可与肿瘤细胞表面靶抗原特异性结合的胞外结构域、跨膜结构域及胞内结构域组成[9]。CAR-T疗法的机制是将编码CAR的基因重组到T淋巴细胞的基因组中并诱导其在细胞表面表达，使 T淋巴细胞具有靶向识别抗原、杀伤肿瘤细胞的作用。CAR-T疗法有望彻底改变MM患者的预后，然而也带来了独特的毒性反应，需要加强监测和及时处理。特殊不良反应如CRS及免疫效应细胞相关神经毒性综合征（ICANS）等受到大量关注，而普遍出现的血液毒性仍缺乏研究，已有研究报道长期严重的血细胞减少与CAR-T治疗后1年生存率有关[10]，故临床医师应提高对血液毒性的重视。目前关于CAR-T治疗后影响血细胞计数恢复的因素尚无一致看法，Jain等[5]认为严重的CRS或ICANS、CRP峰值及铁蛋白峰值与血象延迟恢复有关，Kai等[19]在一项针对CAR-T治疗后持续中性粒细胞减少的研究中发现IL-6峰值、铁蛋白峰值、CRS及ICANS均与血液学毒性无明显关联，因此我们分析了影响血细胞计数完全恢复的可能因素，旨在寻找预测血液毒性的特异性指标，从而对高危患者进行更好的管理。

在这项回顾性研究中，我们研究了初次接受CAR-T治疗患者的造血恢复情况，几乎所有人都出现了血细胞减少，大部分≥3级。在CAR-T细胞输注后90 d时的随访显示，大部分患者血细胞计数可恢复到适当的水平，90 d后因随访数据较少未再纳入统计。单因素分析结果表明外周血铁蛋白峰值、基线WBC及基线HGB为发生影响完全恢复的危险因素，然而多因素分析模型未将这些指标纳入，因此我们暂时认为其作为独立预测完全恢复因素的证据不足。多因素分析显示CRP峰值<68.8 mg/L的患者发生血象延迟恢复的风险为CRP峰值≥68.8 mg/L患者的9.321倍。CRP是临床上常用的炎症指标，且CRP受很多因素影响，如吸烟、体重、血压、血脂、雌激素等[11]，因此该指标与血细胞计数的相关性尚有争议，这一结果尚需更多研究进行验证。基线ANC<1.78×10^9^/L组患者发生血象延迟恢复的风险为基线ANC≥1.78×10^9^/L患者的12.315倍，血小板计数低组发生血象延迟恢复的风险为高组的32.407倍。提示粒细胞与血小板对反应机体造血贮备更敏感，基线ANC或PLT水平较高的患者，或许拥有更好的造血功能，血象恢复的时间更短。CAR-T治疗后的患者外周血中大多都可观察到IL-6升高，IL-6在体内参与多种细胞活动，是一种正性造血调控因子，可刺激造血[12]。但本研究结果显示IL-6峰值>349 pg/L组患者发生血象延迟恢复的风险更高，考虑原因如下：在CAR-T治疗过程中，IL-6被认为是CRS毒性的中心媒介，且IL-6拮抗剂（妥珠单抗）用于治疗CRS已取得可靠的疗效[13]。IL-6的主要来源是巨噬细胞、树突状细胞及淋巴细胞，有人提出T细胞激活的免疫级联反应可能导致了CRS的发生[14]，因此我们推测T细胞及单核-巨噬细胞过度活化分泌的大量毒性细胞因子可能对血细胞有直接破坏作用。本研究两组患者在CRS的发生上无明显差异，但国外有研究表明严重的CRS或ICANS可影响CAR-T治疗后血细胞计数恢复[15]，这可能与CRS评估标准存在一定主观性及观测差异有关，因此我们考虑IL-6对造血恢复的影响可能是通过细胞因子风暴介导。

在输注CAR-T细胞之前，通常先进行淋巴细胞清除化疗，以促进CAR-T细胞在体内的增殖[16]。我们的研究发现，中性粒细胞减少常发生于预处理后，由于预处理化疗与输注细胞时间间隔较短，很难排除预处理对粒系造血的影响，这可能会干扰对粒系造血恢复情况的分析。此外，我们没有捕获所有可能影响恢复的潜在临床变量。将这些变量纳入模型可能会改变本研究中描述的变量的显著性。尽管如此，本研究分析得出的结果或许可为探索CAR-T治疗后血液学毒性的发生机制提供线索。

长期的血小板减少症使患者面临危及生命的出血风险，粒细胞缺乏增加感染的风险[17]，成分输血及相关并发症管理会延长住院时间并增加治疗成本[18]，长期的血细胞减少可能会限制疾病进展时的后续治疗。如果能减少成分输血，可降低治疗费用及改善患者的生活质量，因此促进血象恢复可能会有临床益处。我们的研究或许可用于预测严重的血液毒性，指导临床工作，但在CAR-T治疗前进行临床干预是否会减少血液毒性的发生尚需前瞻性研究进一步验证。
